# Wafer-scale and universal van der Waals metal semiconductor contact

**DOI:** 10.1038/s41467-023-36715-6

**Published:** 2023-02-23

**Authors:** Lingan Kong, Ruixia Wu, Yang Chen, Ying Huangfu, Liting Liu, Wei Li, Donglin Lu, Quanyang Tao, Wenjing Song, Wanying Li, Zheyi Lu, Xiao Liu, Yunxin Li, Zhiwei Li, Wei Tong, Shuimei Ding, Songlong Liu, Likuan Ma, Liwang Ren, Yiliu Wang, Lei Liao, Xidong Duan, Yuan Liu

**Affiliations:** 1 Key Laboratory for Micro-Nano Optoelectronic Devices of Ministry of Education, School of Physics and Electronics, Hunan University, Changsha, 410082 China; 2 State Key Laboratory for Chemo/Biosensing and Chemometrics, College of Chemistry and Chemical Engineering, Hunan University, Changsha, 410082 China

**Keywords:** Electronic devices, Electronic devices

## Abstract

Van der Waals (vdW) metallic contacts have been demonstrated as a promising approach to reduce the contact resistance and minimize the Fermi level pinning at the interface of two-dimensional (2D) semiconductors. However, only a limited number of metals can be mechanically peeled and laminated to fabricate vdW contacts, and the required manual transfer process is not scalable. Here, we report a wafer-scale and universal vdW metal integration strategy readily applicable to a wide range of metals and semiconductors. By utilizing a thermally decomposable polymer as the buffer layer, different metals were directly deposited without damaging the underlying 2D semiconductor channels. The polymer buffer could be dry-removed through thermal annealing. With this technique, various metals could be vdW integrated as the contact of 2D transistors, including Ag, Al, Ti, Cr, Ni, Cu, Co, Au, Pd. Finally, we demonstrate that this vdW integration strategy can be extended to bulk semiconductors with reduced Fermi level pinning effect.

## Abstract (web summary)

Laminated van der Waals (vdW) metallic electrodes can improve the contact of 2D electronic devices, but their scalability is usually limited by the transfer process. Here, the authors report a strategy to deposit vdW contacts onto various 2D and 3D semiconductors at the wafer scale.

## Introduction

Two-dimensional (2D) semiconductors have attracted considerable interest as ultrathin channel materials for transistors^1–6^. With atomically thin body and dangling-bond free surface, 2D semiconductor offers significant potential for ultimate body thickness scaling, which is essential for reducing short channel effect and further extending Moore’s Law^7–11^. However, on the other hand, with ultra-thin body and delicate lattice, achieving high quality metal contact to 2D semiconductors remains a critical challenge^12^. Conventional metallization approaches in microelectronics research (e.g., thermal/e-beam evaporation, sputtering, chemical vapor deposition) are generally “high-energy” fabrication processes based on the vaporization of metal precursor^13–15^. These methods are not necessarily compatible with emerging 2D semiconductors because they usually involve hot metal atoms or clusters bombardment to the contact region, leading to substantial damage via kinetic energy transfer or chemical reaction between the metal atoms and the 2D semiconductors^16–20^. Therefore, strong Fermi level pinning effect is typically observed at metal-2D contact interface with uncontrollable Schottky barrier height and large contact resistance^20–24^, posing an important technological challenge for the investigation of novel physics within 2D semiconductors as well as the realization of high-performance 2D devices.

To avoid the damage during metallization process and to retain the intrinsic properties of 2D contact region, considerable efforts have been devoted to a “low-energy” van der Waals (vdW) integration processes^25–32^. Within this approach, the metal and 2D semiconductor are interacted through weakest vdW force in the contact region, rather than chemical bonding (covalent or ionic), hence could retaining their isolated states without interface disorder. For example, by directly evaporating indium with low melting temperature (157 °C) on the MoS_2_ surface, the intrinsic properties of monolayer MoS_2_ could be well retained during the “low-energy” and gentle deposition process, forming an atomic clean contact between 2D semiconductors and 3D metals with a clear vdW gap in between^25^. Therefore, high-performance monolayer MoS_2_ transistors could be achieved with low contact resistance ~3 kΩ μm (ref. ^25^). Similarly, directly evaporating semimetals^26,33^ (Bi, with melting temperature of 271 °C; Sn, with melting temperature of 232 °C) to monolayer MoS_2_ could minimize the Fermi level pinning effect with negligible contact barrier and lowest contact resistance of 0.12 kΩ μm, approaching that suggested by International Roadmap for Devices and Systems (IRDS)^34^. However, these “low-energy” evaporating processes only work with specific metals (such as In or Bi) and their thermal stability may need further assessment due to low melting temperature.

Alternatively, low-energy vdW contact could be achieved through transferring the pre-fabricated metal electrodes^27–32,35^. By mechanically laminating the flat metal films on the surface of 2D semiconductor, the conventional “high-energy” lithography and metallization processes could be avoided. Hence, the intrinsic physical properties of 2D semiconductors could be well retained, resulting in an ideal metal-2D interfaces and highly tunable Schottky barrier (close to Schottky-Mott limit). However, this method relies on mechanically peeling metals from the pre-deposited substrate, and only a few low-adhesion metals (e.g., Ag, Au, Pt, Pd) could be successfully peeled-off and transferred. For most metals (e.g., Al, W, Ni, Co, Mo, Ti, Ta) used in industry fabrication line, they demonstrate high adhesion force to the pre-deposited substrate and can not be transferred to form vdW contact with 2D semiconductors. To overcome this limitation, recent work directly deposits a thin Se layer on 2D semiconductors as the buffer layer^36^, followed by evaporation of metals on top. By thermally removing the Se buffer, various metals could be vdW contacted with the underlying 2D materials. However, this process still involves high-energy evaporation process by directly depositing Se buffer on 2D lattice, which could impact the intrinsic lattice of 2D material, and the scalability and this method is not explored. Therefore, a damage-free and scalable vdW integration technique between different 3D metals and 2D semiconductors has yet to be demonstrated, posing a critical limitation for the practical application of vdW contact and high-performance 2D transistors.

Here, we report a wafer-scale and universal vdW metal integration strategy that can be readily applicable to different metals and semiconductors. By spin-coating a decomposable poly(propylene carbonate) (PPC) as the buffer layer between metals and 2D semiconductors, the intrinsic properties of 2D monolayers are not impacted. With further thermally annealing, the PPC layer is dry-decomposed into gases and flow away^37,38^, leading to vdW contact between metals and 2D semiconductors. The achieved vdW interface is atomically clean and sharp, as verified through detailed mechanical test, optical microscopy, atomic force microscopy (AFM), scanning electron microscopy (SEM) and high-resolution transmission electron microscopy (HRTEM) characterizations. With this technique, various high-adhesion and industry compatible metals (e.g., Co, Ni, Al, Ti) could be vdW integrated as the contact of 2D transistors, demonstrating highly tunable Schottky barrier and improved electrical performance. Finally, we demonstrate our vdW integration strategy is not only limited to 2D semiconductors, but could be well-extended to bulk semiconductors (such as Ge, GaAs, IGZO, perovskite) with reduced Fermi level pinning effect. Our study not only realizes high-performance 2D transistor through vdW metal contact, but also provides a wafer-scale and general vdW integration process of different metals without uncontrollable peeling and lamination processes.

## Results

### Fabrication processes of the general vdW contact

**Fig. 1:**
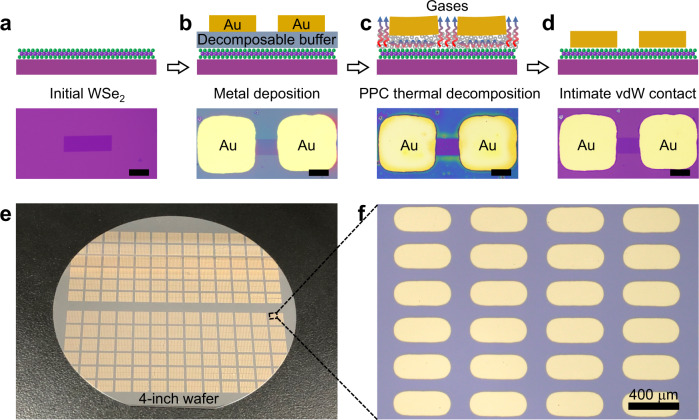
Wafer-scale van der Waals (vdW) integration processes. **a–d** Schematics and optical images of vdW Au-WSe_2_ integration with four steps: WSe_2_ flake prepared on substrate (**a**), poly(propylene carbonate) (PPC) buffer spin-coating followed by metal deposition (**b**), PPC buffer dry-decomposed through annealing (**c**), intimate contact formation between Au and WSe_2_ (**d**). The scale bar is 50 μm. **e**, **f** Optical images of large scale vdW contacts.

Figure 1a–d schematically illustrates the fabrication processes of our vdW metal contact, and the corresponding device optical images are also included. To fabricate the device, WSe_2_ is used as a representative 2D semiconductor, which is synthesized using chemical vapour deposition (CVD) method onto a silicon substrate with 300 nm silicon oxide (Fig. 1a). Next, 450 nm thick PPC polymer is spin-coated on top of WSe_2_. Subsequently, source-drain electrodes (60 nm thick Au) are directly evaporated on top of the PPC using standard thermal evaporation process (Fig. 1b), as detailed in Methods section. Since PPC layer is thick enough, it could work as an effective protection layer to prevent any deposition induced damages to underlayer WSe_2_ or chemical bonding between Au and WSe_2_. We further note the spin-coating of buffer layer is a low-energy process, and is essential to keep the intrinsic properties of 2D monolayers. As shown in Supplementary Fig. 1, the optical and electrical properties of WSe_2_ keeps unchanged before and after spin-coating PPC buffer. Finally, the device is heated at 250 °C for 30 mins under nitrogen environment (Fig. 1c). At this stage, the PPC layer would be totally dry-decomposed into gases and flow away, where the Au are automatically laminated onto WSe_2_ through weak vdW interaction, as schematically illustrated in Fig. 1d. Furthermore, the demonstrated vdW integration process here is compatible with industry process and could be fabricated at wafer-scale, as demonstrated in a 4-inch wafer with over ~25,000 vdW contacts using one batch of fabrication (Fig. 1e, f).

We note the PPC polymer used here is essential for our vdW contact approach owing to its unique properties. It could be dry-decomposed into gases at evaluated temperature (250 °C) without involving solution or residues^37,38^, as confirmed through the XPS (X-ray photoelectron spectroscopy) measurement in Supplementary Fig. 2. Hence, the PPC decomposition process has been widely used in microelectronic applications to create various “air-gaps” structure^39–43^, indicating this process is industry compatible. Since the metals are directly laminated onto the surface of 2D semiconductor, our process could avoid manual transfer process used in previous vdW approach^44^. In addition, the size of individual metal electrode should be <5 mm. For example, if the metal film is continuous (over 1 cm size), the decompose gases will be trapped in the metal film and can not be fully removed, leading to air bubbles or metal film crack, as shown in Supplementary Fig. 3.

**Fig. 2:**
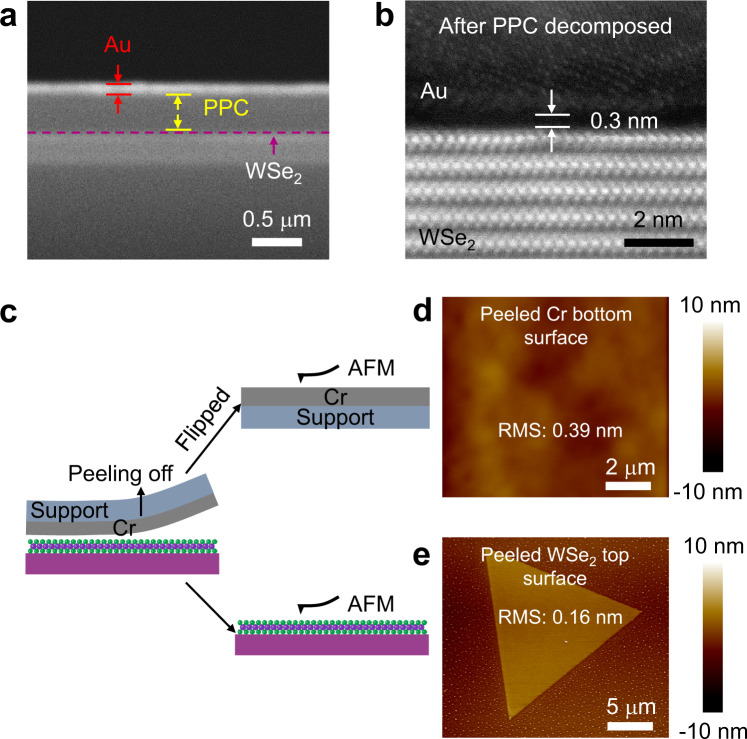
Characterizations of the vdW metal-semiconductor interface. **a** Cross-sectional scanning electron microscopy (SEM) image of WSe_2_/PPC/Au tri-layer structure, purple dashed line represents WSe_2_. **b** Cross-sectional high-resolution transmission electron microscopy (HRTEM) image of the WSe_2_/Au vdW interface after PPC layer decomposed. Atomic sharp and clean metal-semiconductor interface is observed with a vdW gap of ~0.3 nm. **c** The schematic of atomic force microscopy (AFM) sample preparation for characterizing the interface quality in larger area. The as-fabricated Cr/WSe_2_ interface is decoupled by mechanically peeling-off the Cr electrodes, and the bottom surface of Cr electrode could be flipped for AFM measurement. **d** AFM characterization of Cr bottom surface (flipped after peeling), demonstrating a small root-mean square (RMS) of 0.16 nm. **e** AFM characterization of WSe_2_ top surface after peeling-off the Cr electrode, with RMS of 0.39 nm.

To confirm the achieved metal-2D vdW interface is atomically sharp and clean, cross-sectional SEM and HRTEM characterizations are conducted. As shown in Fig. 2a, the as-deposited sample (before PPC decomposition) demonstrates a clear WSe_2_/PPC/Au tri-layer structure, where PPC buffer layer is uniform after metal deposition, preventing any interaction between WSe_2_ and Au during the “high-energy” evaporation process. After the PPC is thermally decomposed, the metal demonstrates intimate contact with the underlayer WSe_2_ without polymer residues or metal-2D chemical interaction, as confirmed in Fig. 2b using HRTEM. Particularly, the WSe_2_ lattice structure is well retained without deposition induced damages and a vdW gap ~0.3 nm is clearly observed (Fig. 2b and Supplementary Fig. 4), indicating the metal is physical laminated on WSe_2_ during PPC decomposition.

Since the HRTEM only characterizes the interface properties of small region (typically tens of nanometers), AFM measurement is further conducted to characterize the interface properties of the whole contact region. Thanks to the weak vdW interaction within the metal-2D contact, the as-fabricated Cr/WSe_2_ vdW interface could be decoupled by mechanically peeling-off the integrated Cr electrodes (Method section), where the bottom surface of Cr film could be flipped for AFM measurement (schematically illustrated in Fig. 2c). As shown in the Fig. 2d, the bottom surface of peeled Cr demonstrates atomic flat surface with a root-mean square (RMS) of 0.39 nm, which replicates the flat surface of the PPC film (Supplementary Fig. 5). In the meantime, the decoupled WSe_2_ demonstrates flat surface with small RMS roughness of 0.16 nm (Fig. 2e), consistent with the as-grown WSe_2_ surface (Supplementary Fig. 6). The flat Cr bottom surface and WSe_2_ top surface (after separating the fabricated interface) is strong evidence of the optimized vdW interface achieved without polymer residues. We note the AFM approach to characterize peeled junction could also be extended to examine other vdW interfaces (e.g., superconductor/insulator, semiconductor/dielectric) with atomic resolution, which could provide more information within two-dimensional area (over 20×20 μm^2^ in our experiment). This could be a complementary approach of commonly used focused ion beam (FIB) cutting followed by cross-sectional TEM, which only provides one-dimensional line information (typically ~100 nm) of the interfacial property.

Furthermore, the intrinsic properties of WSe_2_ are examined through optical measurement. We have compared the Raman and photoluminescence (PL) spectrums of as-grown WSe_2_ and after vdW metal integration. As shown in Supplementary Fig. 7, the ${{{{{{\rm{E}}}}}}}_{1{{{{{\rm{g}}}}}}}^{1}$ Raman peak of WSe_2_ remains at 250.68 cm^−1^ (ref. ^45^) and the PL spectrum remains identical before and after the vdW integration process^46^, indicating our vdW integration process won’t introduce strains or doping effect to the underlayer 2D semiconductor.

### vdW integrating various metals on top of WSe_2_

**Fig. 3:**
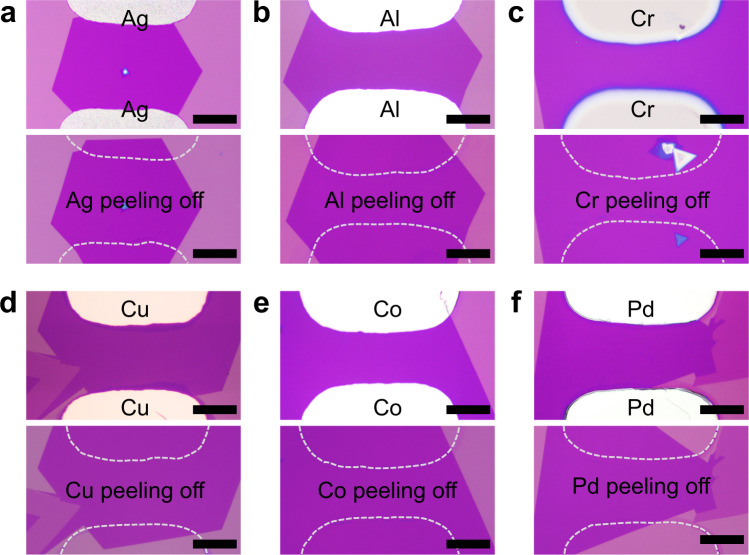
Optical images of WSe_2_ flakes with different contact metals integrated and peeled-off. **a–f** The upper panels show the optical images of different metals integrated on top of WSe_2_ flake, including Ag (**a**), Al (**b**), Cr (**c**), Cu (**d**), Co (**e**), Pd (**f**). In the meantime, the corresponding optical image after peeling-off electrodes are also demonstrated as the lower panels, where the white dash lines are used to highlight the outlines of electrode position. The underlying WSe_2_ nanosheets sustain their original shapes without observable cracks or wrinkles, indicating the weak vdW interaction between different metals with 2D materials. The scale bar is 40 μm.

Our vdW metal-2D contact geometry is realized by decomposing the buffer layer, and is not limited to any specific metal properties. Therefore, it could be theoretically applied to any choices of metals, as long as it can be evaporated on top of the PPC buffer. To demonstrate this, we have fabricated vdW metal-WSe_2_ contact using other metals, including Ag, Al, Ti, Cr, Ni, Cu, Co, Pd, as shown in Fig. 3 and in Supplementary Fig. 8. Importantly, after transistor electrical measurement, all these metals can be further mechanically peeled-off from the WSe_2_, suggesting the weakly interacted contact without chemical bonding between different vdW metals and underlayer WSe_2_. As shown in Fig. 3, the WSe_2_ under contact regions still demonstrate original shape and optical contrast after the metal peeling processes, suggesting the vdW metal integration processes of these metals are damage-free and residue-free, where the 2D semiconductor could retain its intrinsic properties. This is in great contrast to directly deposition of high-adhesion metals (such as Al, Ti, Cr) on top 2D semiconductors, which could form strong chemical bonding and can not be further separated once deposited.

### Electrical performance WSe_2_ transistors

**Fig. 4:**
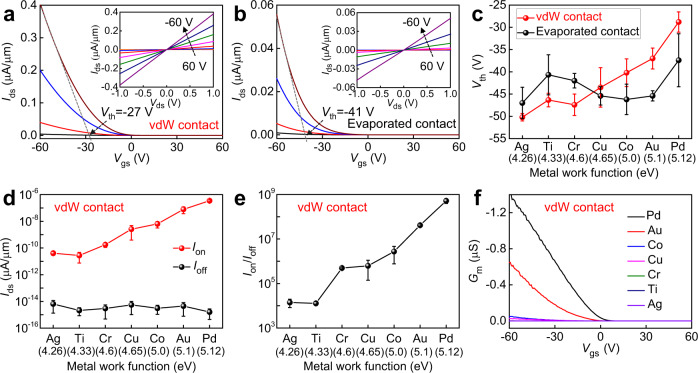
Electrical performance of WSe_2_ transistors using different metal contacts. **a**, **b** The drain source current- gate source voltage (*I*_ds_ − *V*_gs_) transfer curve and drain source current- drain source voltage (*I*_ds_ − *V*_ds_) output curve of WSe_2_ transistors with vdW integrated Pd electrodes (**a**), as well as evaporated Pd electrodes (**b**). **c–e** Extraction of threshold voltage (*V*_th_) (**c**) on-state current densities (**d**), and current on-off ratios (**e**). Error bars in **c**–**e** are determined from the statistical standard deviations of 5 devices. **f** Transconductance (*G*_m_) as a function of *V*_gs_ with different vdW metals.

To demonstrate the robustness of our vdW contact approach, we have measured the electrical properties of the resulting WSe_2_ transistors, where highly doped Si substrate is used as the back gate, 300 nm thick SiO_2_ is used as the gate dielectric, different vdW metals are used as the source drain electrodes. Also, all devices have identical channel length of 50 μm (defined by the stencil mask, see Method), channel thickness of ~1.4 nm (bilayer WSe_2_), and varied channel width from 90 μm to 135 μm (depending on the flake size of grown WSe_2_). For comparison, WSe_2_ transistors fabricated using conventional evaporated contacts are also measured, where identical channel material (WSe_2_ from same batch of CVD growth) and same device structure are used for fair comparison. As shown in Fig. 4a, b, using Pd as the contact metal, devices with both contact approaches exhibit p-type drain source current- gate source voltage (*I*_ds_-*V*_gs_) transfer characteristics with linear drain source current- drain source voltage (*I*_ds_-*V*_ds_) output curve. The major difference is their driving current, where the extracted on-state current (*V*_gs_ of -60 V, *V*_ds_ of 1 V) of vdW contact is 0.5 μA μm^−1^, 7 times higher compared to control sample with deposited contacts. We note the current density (in Fig. 4a, b) is largely limited by the long channel length and small bias voltage, which could be further boosted using shorter channel, thinner gate dielectric or higher bias voltage (Supplementary Fig. 9).

Furthermore, we have systematically measured vdW integrated device as well as directly deposited device using seven different contact metals (Ag, Ti, Cr, Cu, Co, Au, Pd). As shown in Fig. 4c–f and in Supplementary Fig. 10, the electrical properties of vdW-contacted device are highly sensitive to the metal work functions, where higher work function metals lead to larger on-state current densities (*I*_on_), higher on-off ratios (*I*_on_/*I*_off_), more positive threshold voltage (*V*_th_) and smaller subthreshold swing (*SS*), consistent with band alignment theory between metal work function and the valance band of p-type WSe_2_. In contrast, for devices contacted with directly evaporated metals, their electrical parameters are relatively insensitive with metals (Supplementary Fig. 10), indicating the strong Fermi level pinning effect during metal evaporation process^18,22–24^. In particular, the two-point transconductance *G*_m_ (with different metals contact) is extracted according *I*_ds_-*V*_gs_ transfer curve, as shown in Fig. 4f. The extracted highest *G*_m_ is 1.4 μS for vdW contacted (Pd) device, over 7 time higher than control device with evaporated Pd (0.18 μS). Since same channel material is used, the observed better electrical properties could be largely attributed to the optimized contact using our vdW integration with reduced Fermi level pinning effect, consistent with previous reports^25–27^.

To further confirm the optimized contact in our vdW geometry, we have measured the contact resistance (*R*_C_) of different vdW metals using the transfer length method. As shown in Supplementary Fig. 11, low *R*_C_ of 5.3 kΩ·μm and 10.2 kΩ·μm is achieved by vdW integrating high work function Pd and Au, respectively; while vdW integrating low work function metals (Ag and Ti) yields much larger *R*_C_ of 27.5 MΩ·μm and 3.9 MΩ·μm, respectively. Therefore, the metal-dependent electrical properties (such as mobility or *I*_on_) could be attributed to change of *R*_C_ using different metal and the switching between contact-limited and channel-limited regimes^47^. We also note reducing the *R*_C_ to the IRDS target^34^ has been a major focus in 2D research community recent year, and vdW contact geometry has demonstrated promising potential for realizing this target. Currently, the *R*_C_ achieved in our method (5.3 kΩ·μm) is still much higher than *R*_C_ of previous vdW contact^48,49^, which could be largely attributed to the thick gate dielectric and inferior quality of channel material, and could further improved using thinner dielectrics, as shown in Supplementary Fig. 12. Besides *R*_C_, the Schottky barrier height (*Φ*_SB_) could be also measured by varying the measurement temperature (from 300 K to 100 K), and the extracted *Φ*_SB_ are 116 meV, 103 meV, 83 meV, 53 meV, 36 meV, for Ag, Ti, Ni, Co, Pd, respectively (Supplementary Fig. 13). We note although the measured barrier height is linear related to the metal work functions, the slope (*Φ*_SB_ vs. metal work function) is far from unity and n-type WSe_2_ is still not realized even using low-work function metals, which could be attributed to CVD grown WSe_2_ with large intrinsic defects and strong p-type doping effect.

### vdW contact for other 2D and 3D bulk semiconductors

**Fig. 5:**
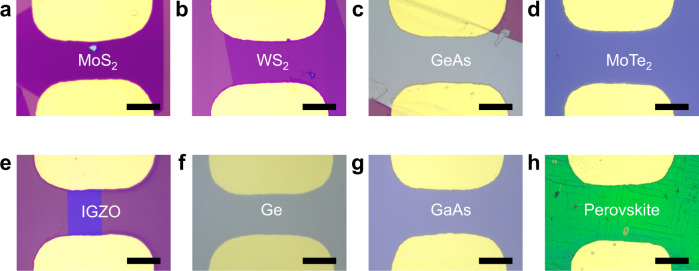
vdW contact integration for other 2D semiconductors and 3D bulk semiconductors. **a–d** Optical images of Au electrode pair integrated on top of chemical vapour deposition (CVD)-grown MoS_2_ flake (**a**), CVD-grown WS_2_ flake (**b**), mechanically exfoliated GeAs flake (**c**) and CVD-grown MoTe_2_ thin-film (**d**). **e–h** Optical images of Au electrode pair integrated on top of amorphous oxide IGZO (**e**), group IV Ge (**f**), group III–V compound GaAs (**g**), and perovskite microcrystal CsPbX_3_ (**h**). The scale bar is 40 μm.

This simple vdW contact approach is not only limited to WSe_2_, but could be well-extended to other 2D semiconductors or 3D bulk semiconductors to achieve vdW metal-semiconductor junction, and to avoid the metal induced damages to the contact region. As shown in Fig. 5, various transistors using this vdW metal contact have been successfully achieved including 2D MoS_2_ (Fig. 5a), WS_2_ (Fig. 5b), GeAs (Fig. 5c), MoTe_2_ (Fig. 5d), as well as 3D amorphous oxide IGZO (indium-gallium-zinc-oxide, Fig. 5e), group IV Ge (Fig. 5f), group III–V compound GaAs (Fig. 5g), perovskite microcrystal CsPbX_3_ (Fig. 5h). In particular, the electrical properties of 2D p-type semiconductor MoTe_2_ and 3D n-type semiconductor IGZO transistors have been systematically measured using different vdW contact metals with distinct work functions. As shown in Supplementary Fig. 14, the vdW contacted devices demonstrate tunable electrical behavior, where the on-state current and *V*_th_ are depended on the work function of vdW metals, suggesting the reduced Fermi level pinning effect is a general trend for different semiconductors. This is in great contrast to MoTe_2_ and IGZO devices using directly deposited metals, where the electrical behavior is relatively insensitive to the metal work functions, demonstrating strong Fermi level pinning effect, as shown in Supplementary Fig. 14.

In addition, we note the demonstration of vdW metal integration approaches to 3D bulk semiconductor may open up a new avenue for a variety of well-studied semiconductors with delicate lattice, including compound semiconductors, ultra-thin organic thin films/crystals, and the halide perovskite materials. Such materials are usually not stable under high temperature, not compatible with traditional micro-fabrication processes or are highly prone to degradation during the electrode deposition process. Besides the advantages of device fabrication, vdW integration may provide reduced pinning contacts for other 3D semiconductor (similar as IGZO), which could be used to design devices that require either low contact barrier for efficient carrier injection (e.g., transistors) or high contact barrier for efficient charge separation (e.g, Schottky-based photodetectors).

## Discussion

In summary, we have demonstrated a scalable and universal vdW integration method that can be readily applicable to different metals and semiconductors. Utilizing PPC buffer layer, various metal electrodes could be physically contacted with the 2D semiconductor through a simple dry-annealing process, yield an atomic clean vdW interface. Compared to previous vdW integration (using metal transfer process), our strategy here does not rely on the metal peeling process, and thus could be used to vdW integrate different metals. More importantly, the direct evaporation process on PPC buffer could avoid the air-bubbles or wrinkles during metal transfer process with higher alignment resolutions, especially in wafer-scale. Therefore, various industry-compatible metals could be vdW integrated as the contact of 2D transistors, including Ag, Al, Ti, Cr, Ni, Cu, Co, Au, Pd, exhibiting improved electrical performance depending on the metal work functions.

Furthermore, this dry-decomposed process can be extended to other buffer layers with distinct properties (e.g, polyphthalaldehyde (PPA), polyethylene carbonate, hyperbranched polymers (HB560)), which have been demonstrated to fabricate uniform air-gaps structure in different temperature range. Finally, we have also demonstrated our vdW integration strategy could be well-extended to different 2D semiconductors as well as bulk semiconductors (Ge, GaAs, IGZO, perovskite) with weakly coupled vdW interface. These demonstrations may intrigue implications for bulk semiconductors that are previously plagued by the high energy metallization process and ill-defined metal-semiconductor contacts, enabling new device structures or high-performance devices.

## Methods

### Preparation of CVD-grown 2D semiconductors, perovskite microcrystals, and IGZO thin film

For preparation of WSe_2_, WS_2_, MoS_2_, powder and a piece of SiO_2_/Si substrate (300 nm SiO_2_) were put into the center and downstream end of tube furnace, respectively. After purging out the air and water vapor inside the tube furnace by argon (Ar), large-scale monolayer or bilayer 2D materials were synthesized (at 1170 °C for WSe_2_, 1200 °C for WS_2_, 650 °C for MoS_2_) for 5 min with Ar flow rate of 80 sccm (ref. ^50^).

For fabrication of MoTe_2_ thin-film, about 3 nm-thick Mo films were firstly evaporated on SiO_2_/Si substrates (300 nm SiO_2_) through e-beam evaporation. Subsequently, after purging out the air and water vapor inside the tube furnace by Ar, the Mo film are tellurized at the temperature of 550 °C. Finally, about 7 nm-thick MoTe_2_ thin-film was synthesized in the downstream end of the tube furnace^51^. For fabrication of perovskite microcrystals, single-step CVD growth of cesium lead halide (CsPbX_3_) microcrystal was conducted^52^.

For preparation of amorphous IGZO, 10 nm-thick channel thin film was deposited on SiO_2_/Si substrates (300 nm SiO_2_) by using RF magnetron sputtering method. During the sputtering process, the working power was controlled at 50 W under the vacuum pressure of 0.7 Pa with Ar flow rate of 15 sccm.

### Fabrication processes of vdW integration and electrodes peeling-off

Firstly, the poly(propylene carbonate) (PPC) precursor solution with a concentration of 10 wt% was obtained by dissolving PPC polymer into anisole at room temperature. The resulting precursor solution was spin-coated at a speed of 5000 rotations per minute (r.p.m). Then the samples were annealed on the preheated hot plate at 120 °C for 2 min to form a 450 nm-thick film. Next, 60 nm-thick electrodes were evaporated by standard thermal evaporation process under vacuum (pressure ~5 × 10^−4^ Pa), with the assistance of stencil mask. Finally, the samples were placed on a hot plate in the glove box, and annealed at 250 °C for 30 mins to entirely decompose the PPC film. To peel-off the integrated metal films, PPC precursor solution was spin-costed on top of vdW metal-2D junction at a speed of 3000 r.p.m to wrap the metal electrodes inside. Then the samples were annealed on the preheated plate at 120 °C for 2 min. At last, PPC film is mechanically peeled-off from 2D materials by using Scotch tape, together with the metal electrodes wrapped. We note that the steel-based stencil mask is used in our process, which typically have a resolution >10 μm. This resolution could be further improved to sub-μm using polymer-based stencil mask^53^.

### Electrical measurement and material characterization

The electrical measurements were collected in a Lakeshore PS-100 cryogenic probe station at room temperature in vacuum (pressure ~5 × 10^−5^ mTorr), using Keysight B2900A source measurement unit (SMU). Raman and PL spectrum measurement (Renishaw invia-reflex) was conducted by using confocal microscope with 488 nm laser as the excitation source.

## Supplementary information

Supplementary Information

## Data Availability

Relevant data supporting the key findings of this study are available within the article and the Supplementary Information file. All raw data generated during the current study are available from the corresponding authors upon request.
