# Supplementary material for: Wafer-scale and universal van der Waals metal semiconductor contact
Source: Nat Commun. 2023 Feb 23;14:1014. doi: 10.1038/s41467-023-36715-6 (PMC9950472; doi:10.1038/s41467-023-36715-6)
Supplement: Supplementary file 1 — Supplementary Information [file 41467_2023_36715_MOESM1_ESM.pdf]

Supplementary Information for

Wafer-scale and universal van der Waals metal semiconductor contact

Lingan Kong[1, #], Ruixia Wu[1, 2, #], Yang Chen[1], Ying Huangfu[2], Liting Liu[1], Wei Li[2], Donglin Lu[1], Quanyang Tao[1], Wenjing Song[1], Wanying Li[1], Zheyi Lu[1], Xiao Liu[1], Yunxin Li[1], Zhiwei Li[1], Wei Tong[1], Shuimei Ding[1], Songlong Liu[2], Likuan Ma[1], Liwang Ren[1], Yiliu Wang[1], Lei Liao[1], Xidong Duan[2], Yuan Liu[1*]

[1]Key Laboratory for Micro-Nano Optoelectronic Devices of Ministry of Education, School of Physics and Electronics, Hunan University, Changsha 410082, China.

[2]State Key Laboratory for Chemo/Biosensing and Chemometrics, College of Chemistry and Chemical Engineering, Hunan University, Changsha 410082, China.

[#] These authors contributed equally to this work.

[*]Corresponding author. Email: yuanliuhnu@hnu.edu.cn

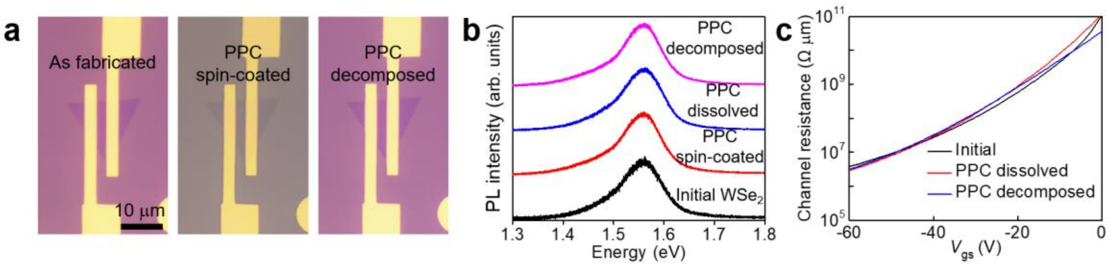

Supplementary Fig. 1. Influence of the buffer layer integration processes on monolayer WSe_2 channel. (**a**) Optical images of the as-fabricated WSe_2 transistor (left), with poly(propylene carbonate) (PPC) buffer layer spin-coated (middle), and PPC buffer dry-decomposed (right). (**b**) Photoluminescence (PL) spectrums of as-fabricated WSe_2, after PPC spin-coated, after PPC dissolved, and after PPC dry-decomposed. The PL peak remains identical during these processes. (**c**) The corresponding channel resistance against gate voltage (V_{gs}) of WSe_2 transistors with consistent electrical properties observed, indicating the PPC integration and removing processes won't change the intrinsic properties of monolayer WSe_2.

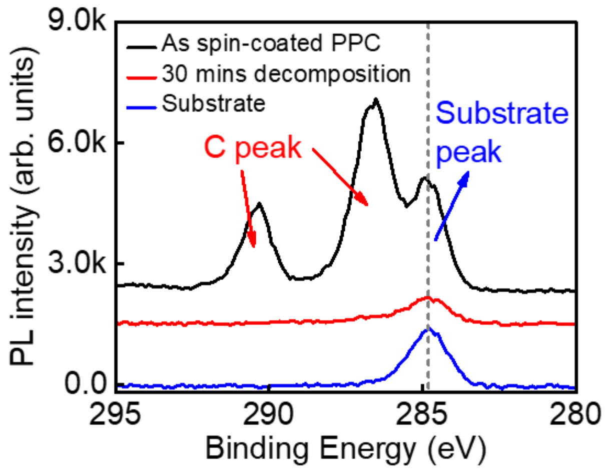

Supplementary Fig. 2. Characterizations of PPC residues. X-ray photoelectron spectroscopy (XPS) measurement of as spin-coated PPC (black), after thermally

annealing (red), and the bare substrate (blue). After thermally annealing, the dominated carbon peaks at 286.6 eV and 290.3 eV disappear, with only the substrate peak left (284.8 eV), indicting the carbon residues could be removed.

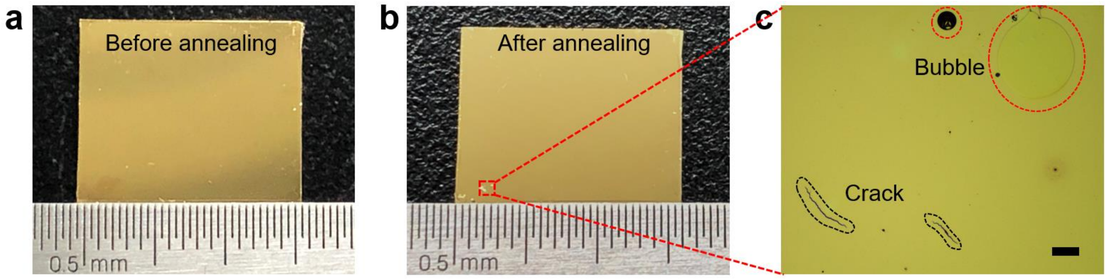

Supplementary Fig. 3. Images of a larger Au film after PPC decomposition. (**a**) Photograph of Au/PPC/WSe_2 using continuous Au film (without stencil) over 1 cm. (**b, c)** Photograph and optical images of the corresponding device after PPC decomposition, where film cracks or air bubbles can be observed. The scale bar in (**c**) is 200 μm.

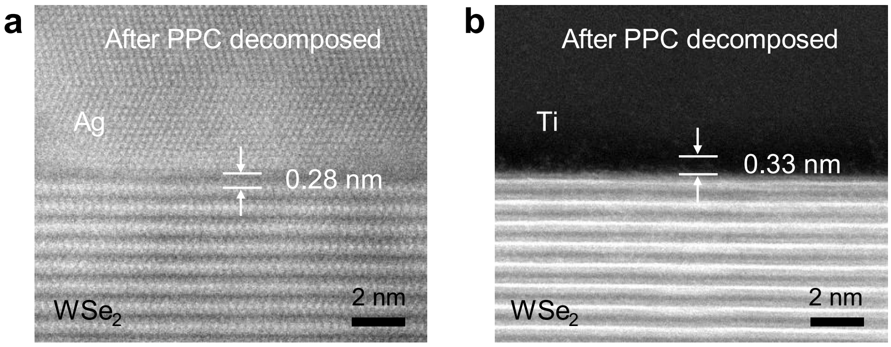

Supplementary Fig. 4. Characterizations of the van der Waals (vdW) metal-semiconductor interface. (**a, b**) Cross-sectional high-resolution transmission electron microscopy (HRTEM) images of the WSe_2/Ag (**a**) and WSe_2/Ti (**b**) interface after PPC

layer decomposed. Atomic sharp and clean metal-semiconductor interface is observed with a vdW gap of ~0.28 nm and ~0.33 nm, respectively.

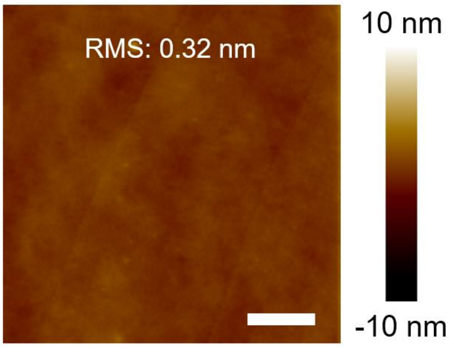

Supplementary Fig. 5. Characterization of PPC surface morphology. Atomic force microscopy (AFM) image of PPC surface (spin-coated on silicon substrate), demonstrating a small root-mean square (RMS) roughness of 0.32 nm. The scale bar is 6 μm.

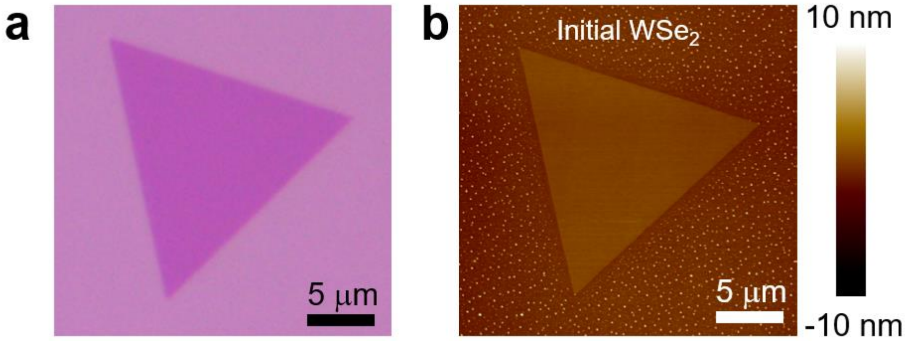

Supplementary Fig. 6. Optical image and AFM image of chemical vapour deposition (CVD) grown WSe_2 flake. (**a**) Optical image of a CVD grown WSe_2 bilayer. (**b**) The corresponding AFM image, demonstrating a small RMS roughness of 0.1 nm.

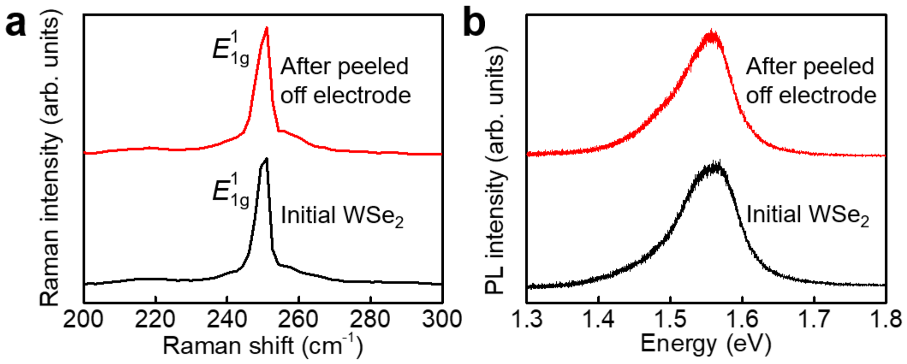

Supplementary Fig. 7. Raman and photoluminescence spectrums with initial WSe_2 and after vdW integration. (**a**) Raman spectrum of as-grown WSe_2 and after Au metals (60 nm thick) vdW integrated, the peak at 250.68 cm^{-1} remains identical before and after peeling-off the electrodes. (**b**) The photoluminescence spectrum of as-grown WSe_2 nanosheets and after peeling-off the electrodes.

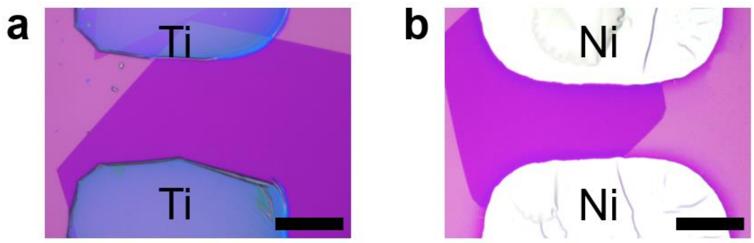

Supplementary Fig. 8. Optical images of WSe_2 transistors with different metals vdW contact. (**a**) Optical image of WSe_2 transistors with Ti contact metal. (**b**) Optical image of WSe_2 transistors with Ni contact metal. The scale bar is 40 μm.

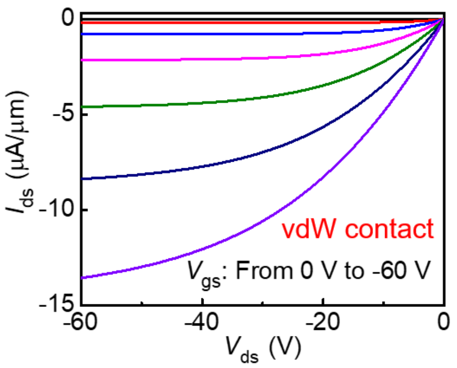

Supplementary Fig. 9. Electrical property of WSe_2 transistor with Pd metals vdW contact. Drain source current- drain source voltage (I_{ds}-V_{ds}) output curves of bilayer WSe_2 transistor using vdW Pd electrodes, the channel length is 50 μm, the V_{ds} is from 0 to -60 V, and gate source voltage (V_{gs}) is from 0 to -60 V.

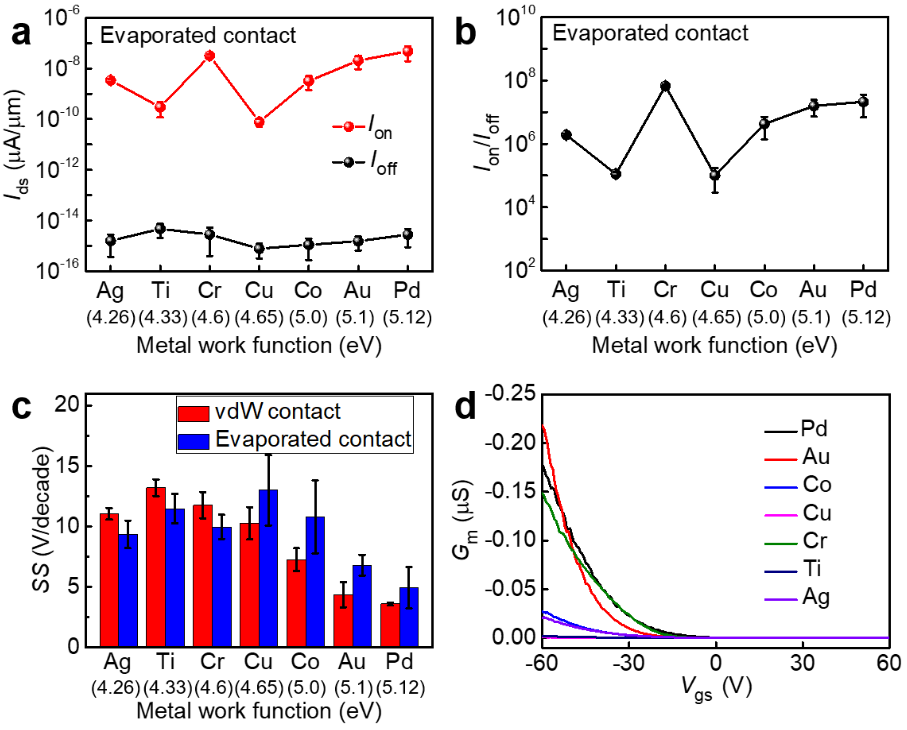

Supplementary Fig. 10. Electronic properties of WSe_2 transistors. (**a, b**) on-state current (I_{on}) and off-state current (I_{off}) (**a**), I_{on}/I_{off} ratio (**b**) of WSe_2 transistor with

evaporated electrodes. (**c**) Subthreshold swing (*SS*) with vdW integrated (red) and evaporated electrodes (blue). (**d**) Extracted transconductance (G_m) as a function of V_{gs} using evaporated electrodes. Error bars in these figures are determined from the statistical standard deviations of 5 devices.

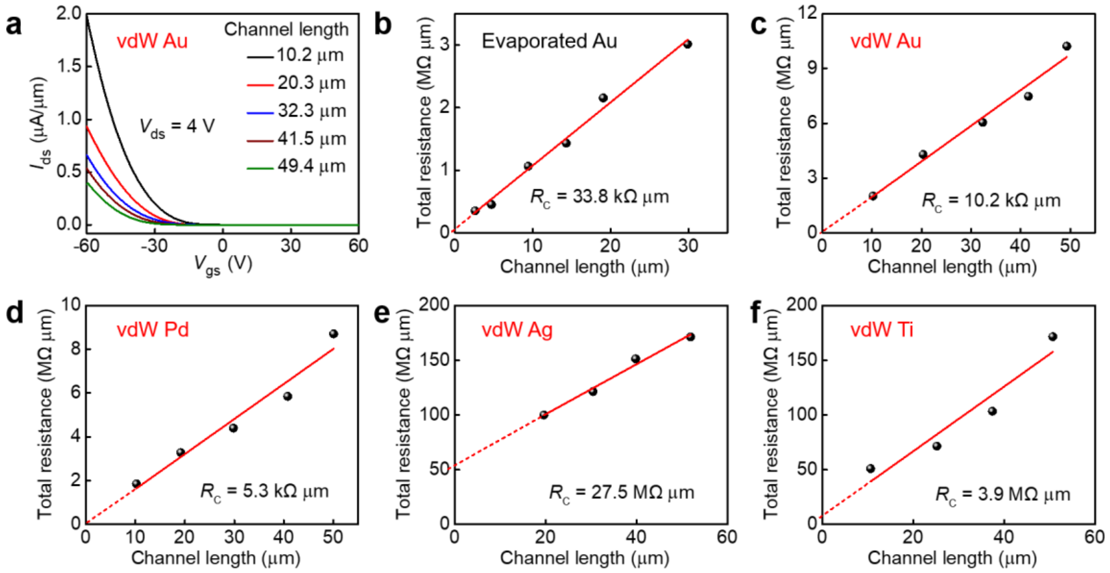

Supplementary Fig. 11. Contact resistance (R_C) of WSe_2 transistors with vdW integrated electrodes. (**a**) Drain source current- gate source voltage (I_{ds}-V_{gs}) transfer curves of WSe_2 transistor using vdW Au electrodes with different channel lengths. (**b-e**) R_C extraction of bilayer WSe_2 transistors with vdW integrated Au (**b**), Pd (**c**), Ag (**d**), and Ti (**e**). The R_C of WSe_2 transistors is extracted to be 10.2 kΩ·μm, 5.3 kΩ·μm, 27.5 MΩ·μm, and 3.9 MΩ·μm, respectively.

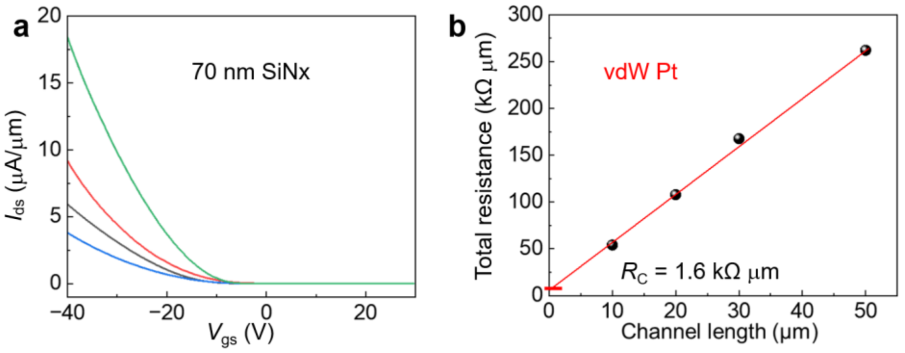

Supplementary Fig. 12. R_C of WSe_2 transistors with high-k dielectric. (**a**) I_{ds}-V_{gs} transfer curves of WSe_2 transistors (70 nm thick SiNx as the back gate dielectric) using vdW Pt electrodes with different channel lengths. (**b**) Total resistance as a function of channel length, yielding a R_C~1.6 kΩ·μm.

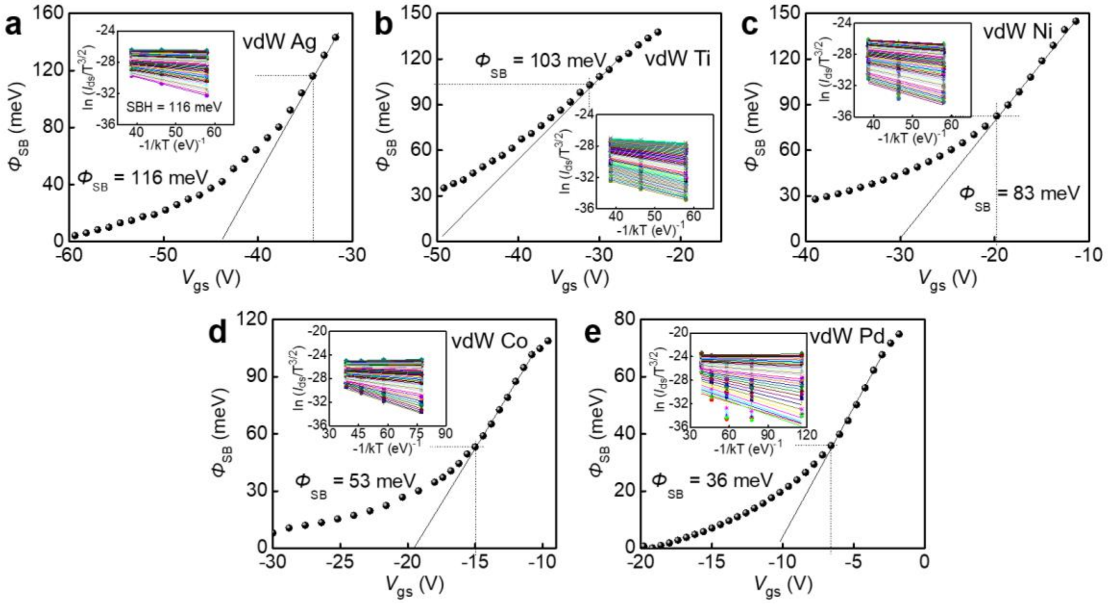

Supplementary Fig. 13. Schottky barrier height (Φ_{SB}) extraction using different vdW metals. (**a-e**) The extracted barrier is plotted as a function of gate voltage and the effective Schottky barrier height is defined by flat band method. Insets are the Arrhenius plots between -1/kT and ln($I_{ds}/T^{1.5}$). The bias voltage for Φ_{SB} extraction throughout (**a-e**) is fixed at 100 mV.

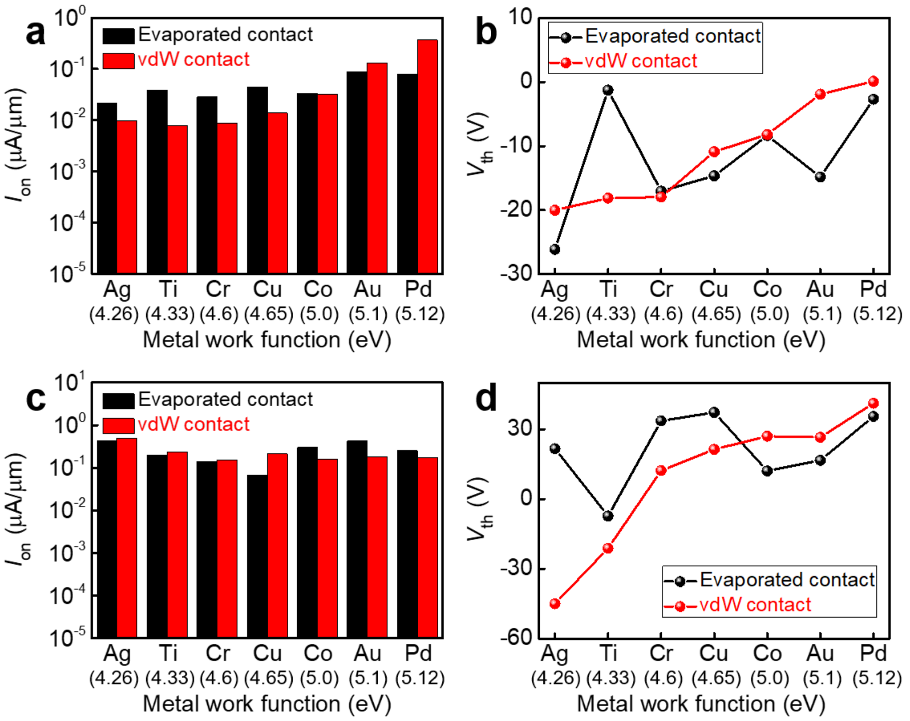

Supplementary Fig. 14. Electrical measurement $MoTe_2$ and IGZO (indium-gallium-zinc-oxide) transistors. (**a, b**) I_{on} densities (**a**) and threshold voltage (V_{th}) (**b**) of p-type $MoTe_2$ transistors using different metal integration approaches, where the vdW integrated devices is more sensitive to the metal work function. (**c, d**) I_{on} densities (**d**) and V_{th} (**e**) of n-type IGZO transistors fabricated through different metal integration approaches.
